# The adaptation process of a safety planning intervention to promote connectedness and reduce distress in Diné adolescents and adults during the COVID-19 pandemic

**DOI:** 10.3389/fpubh.2024.1376686

**Published:** 2025-01-16

**Authors:** Joshuaa Dominic Allison-Burbank, Vanessa Begaye, Kyann Dedman-Cisco, Lisa Jim, Shannon Archuleta, Renae Begay, Lacey Howe, Emily E. Haroz

**Affiliations:** Department of International Health, Center for Indigenous Health, Johns Hopkins Bloomberg School of Public Health, Baltimore, MD, United States

**Keywords:** COVID-19, safety planning, mental health, American Indian, stress

## Abstract

**Introduction:**

Indigenous connectedness is an impetus for health, well-being, self-confidence, cultural preservation, and communal thriving. When this connectedness is disrupted, the beliefs, values, and ways of life that weave Indigenous communities together is threatened. In the Spring of 2020, the COVID-19 virus crept into Tribal Nations across the United States and exacerbated significant health-related and educational inequities. The first case of COVID-19, or *Dikos Ntsaaígíí-19* in the *Diné* (Navajo) language, arrived in the Southwest in March 2020. The virus quickly spread amongst Diné communities and contributed to immediate school closures. These closures created significant disruption to connectedness on the Navajo Nation.

**Methods:**

As part of the Community Based Participatory Research process, our team worked with a Community Advisory Board (CAB) to facilitate a longitudinal cohort study titled “Project SafeSchools” and, most critically, to culturally adapt mental health interventions to be implemented as a part of this study. This paper describes the process our team used to select, adapt, and test Safety Planning and Caring Contacts interventions to reduce elevated rates of depression, anxiety, and suicidal ideation amongst Diné adolescents and adults. CBPR was the primary approach used to engage with Navajo Nation communities and the adaptation process was guided by a scoping study of frameworks for adapting public health evidence-based interventions (EBI) and was guided by the cultural adaptation process. Our team met virtually several times in 2021 and 2022 as the parent launched and as the pilot randomized clinical trial called “+Connection is Medicine” started. When Safety Planning and Caring Contacts messaging was selected, the study team, which consisted primarily of Navajo research personnel led by a Navajo community-based principal investigator (PI) and allied PIs with extensive experience in implementation science, infectious disease prevention, and CBPR, were adapted and presented to CAB members. This CAB also included a youth advisory council who participated in the field testing and further adaptation process.

**Results:**

The use of the CAB allowed for a collaborative workgroup effort to examine the feasibility and acceptability of using safety planning to help reduce suicide risk factors with Navajo adolescents and adults. Most importantly, this CAB collaborative with researchers to further tailor safety plan intervention materials to align with Navajo values related to connectedness to relatives, community, mental health resources, and the land. In addition, the caring contacts messaging was aligned with safety plans to provide culturally sensitive messages that would be shared with randomized participants.

**Discussion:**

Mental health stigma is highly common in reservation-based communities. In Indigenous communities, mental illness has association with not living well or not living culturally aligned further complicates the likelihood of at-risk community members contacting resources available in their communities. By using an Indigenous approach that restores connectedness, and reminds participants of their belonging, +CiM researchers were able to develop enhanced versions of safety plans to use in their pilot randomized controlled trial.

## Introduction

“Before me peaceful, behind me peaceful, under me peaceful, over me peaceful, all around me peaceful.” ~ A Diné Teaching.

**Figure 1:**
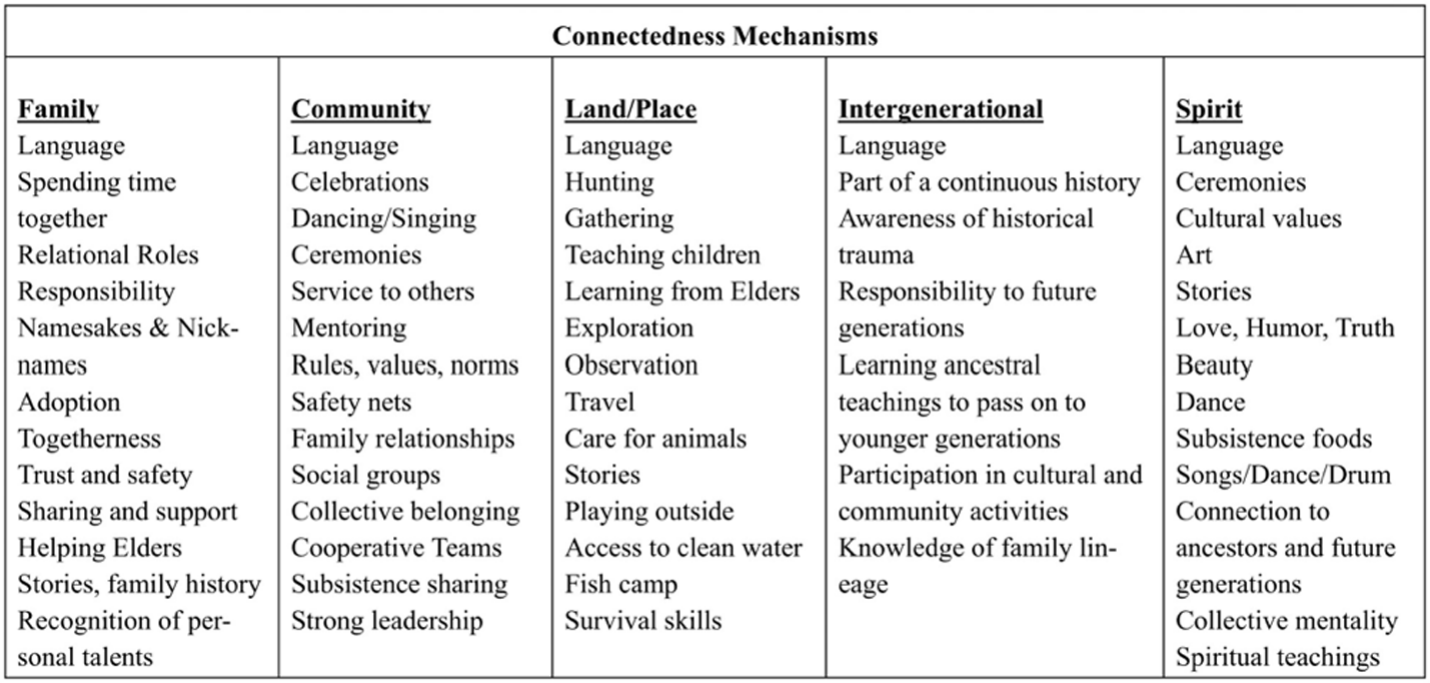
Mechanisms of Indigenous Connectedness offered by Ulrich.

Dr. Jessica Ulrich, an Inupiaq researcher, presents a context for this paper – “Indigenous peoples are not trapped in a traumatic past (1, p.1).” We start this paper with this perspective to purposefully celebrate Indigenous resilience. Ullrich (1) goes on to describe how Indigenous people have always overcome hardships and provides a framework for how to represent the interplay of Indigenous connectedness and resilience. It is through this resilience that we identify vital protective factors of Indigenous flourishment as we strive to build peaceful and healthier Indigenous communities while also supporting these communities after the COVID-19 pandemic. A prominent feature of healthy Indigenous communities is connectedness, evidenced by the observation of thriving Indigenous communities. The communities that maintain their old ways of living and knowledge are often the communities that demonstrate higher resilience in times of hardship. It is also these communities that model how Indigenous values can serve as protective factors for well-being. To “live in a good way” means that the Indigenous person knows who they are and where they come from. They also live in balance with their children, partners, family, community, and the earth. Ulrich’s Indigenous Connectedness Framework depicts essential connectedness mechanisms that are unique to Indigenous peoples: Family, Community, Land/Place, Intergenerational, and Spirit (see Figure 1 for connectedness mechanisms). Indigenous connectedness is an impetus for health, mental well-being, self-confidence, stability, cultural preservation, self-identity and communal thriving. When this connectedness is disrupted, the glue that holds Indigenous communities together is threatened. We utilized this framework as way to address the mental health stigma common in Indigenous communities by conceptualizing a traditional value related to belonging within a family and community, which has proven to be protective of mental health status (2–4). This approach allowed the +Connection is Medicine research team to think of mental health solutions from an Indigenous perspective and to tailor interventions designed to foster connectedness and restore belonging for adolescents and adults impacted by the COVID-19 pandemic.

## Background

In the Spring of 2020, the COVID-19 virus crept into Tribal Nations across the United States (U.S.) and exacerbated significant health-related and educational inequities. Tribal Nations were tasked with protecting and advocating for their sovereignty and demonstrating self-determination to help ensure the survival of Indigenous peoples and their ways of knowing. Early in the pandemic, mortality was the highest amongst American Indian and Alaska Native (AI/AN) people (5). During this time, most public schools across the U.S. stopped in-person learning in the Spring of 2020 to reduce the transmission of the virus among child populations. The first case of COVID-19, or *Dikos Ntsaaígíí-19* in the *Diné* language, arrived in the Southwest in March 2020. Amongst the Navajo Nation communities the virus spread quickly resulting in immediate school closures across the reservation. During the 2020–2021 academic school year, most schools on the reservations remained closed for in-person learning, and many switched to virtual learning, as a result of Tribal Nations having sovereignty over their lands. The switch entirely to online learning was challenging and has had lasting impacts on child learning (25, 26). Schools on the reservation were known to provide critical nutritional, physical, and social–emotional support to students and families before the pandemic. The reservation-based schools did not only focus on the youths’ academic instructions but served as an access point for nutritious breakfast and lunch, healthcare, and connection to peers and other adults who provide critical social support to the students. While schools and families mounted tremendous efforts to overcome these disruptions (e.g., delivery of meals, school buses equipped with Internet hotspots and delivery of school supplies to students), school closure contributed to a reduction in access to critical resources especially those who were in need most (6, 7). In response to this our team of Indigenous and allied researchers pivoted to serving the community by conducting formative research which provided foundational knowledge on the mental health and psychosocial impacts of COVID-19 and school closures on Diné youth and adult wellbeing.

In the Spring of 2021, our team launched the “Project SafeSchools” (PSS), a prospective cohort study on the Navajo Nation and another Southwest Tribe. The study aims were to: (1) understand the barriers and facilitators to school re-opening and in-person school attendance from the perspective of multiple stakeholders in Diné and Apache communities; and (2) evaluate the educational, social, emotional, physical, and mental health impacts of returning to in-person learning for caregivers and youth ages 4–16 (8). This study was approved by the Navajo Nation Health Research Board, agency councils, and local chapter governments. We also had the support from the Navajo Nation Department of Diné Education and the local Indian Health Service units.

As part of the Indigenous Community Based Participatory Research (ICBPR) framework, our community-based study team leads worked with local Community Advisory Boards (CABs) which consisted of tribal community members and partners to help in the design of the PSS study and provide ongoing feedback (9). Further, the ICBPR included frequent engagement with tribal governing agencies, such as chapter houses, tribal councils, and tribal public health offices (10). Preliminary results from the initial baseline surveys indicated high levels of distress among caregivers and their youth. Our CABs urged action and in response, our team pivoted to selecting and adapting evidence-based interventions to address distress in our study population. Thus, a pilot randomized controlled trial called +Connection is Medicine (+CiM) on the Navajo Nation was created. This paper aims to describe the process our team used to select, adapt, and test evidence-based interventions that can be leveraged to promote connectedness and reduce stress for the families enrolled in the PSS cohort.

## Methods

The primary objective of the +CiM intervention was to reduce elevated rates of depression, anxiety, and suicidal ideation in our parent study pool through the adaptation of Safety Planning and Caring Contacts interventions which have successfully been used in communities that have experienced highly traumatic events, including veterans and Indigenous people. A key component of this adaptation was the culturally tailoring of this intervention to prioritize Indigenous connectedness as a conceptual framework. In this paper, we focus on the adaptation process that took place on the Navajo Nation, specifically, within the communities of Shiprock, NM, Chinle, AZ, and Tuba City, AZ. The adaptation process primarily focused on the experiences of reservation-based Navajo families. The overall study was approved by the Johns Hopkins Bloomberg School of Public Health Institutional Review Board (IRB #00020570) and the Navajo Nation Human Subjects Research Review Board (NNR-22.445).

## Science of adaptation

**Figure 2:**
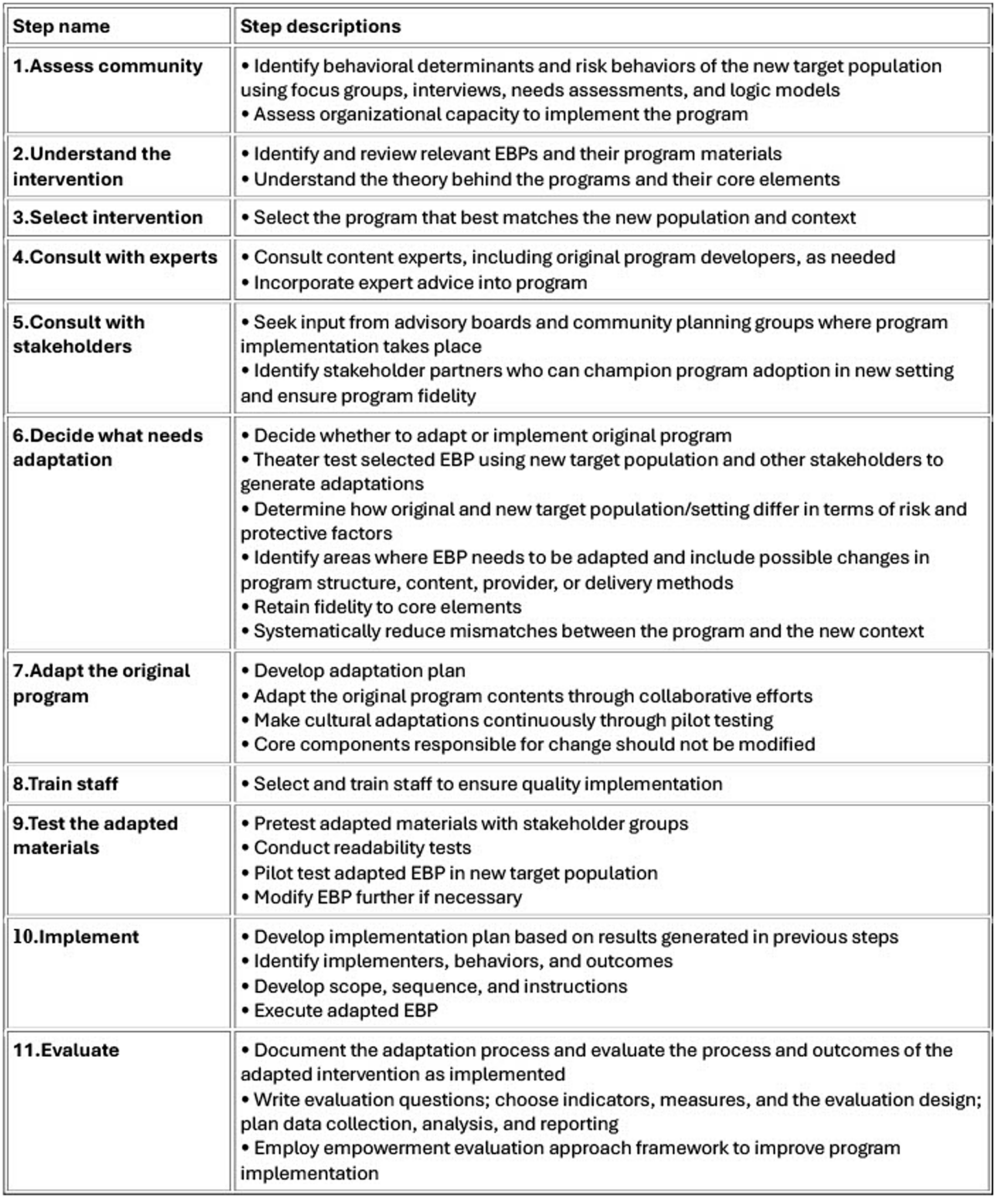
Stages of Implementation Science.

Furthermore, we used the steps identified by Escoffery et al. (11) to organize how our research team adapted our +CiM intervention to promote connectedness and reduce distress for vulnerable study participants. The adaptation process is based on a scoping study of frameworks for adapting public health evidence-based interventions (EBI) and was guided by the cultural adaptation process of Ward et al. (12). This approach offers researchers strategies on how to organize adaptation of existing EBIs and has been commonly used by public health teams. The stages include the following: (1) assess the community, (2) understand the EBI(s), (3) select the EBI, (4) consult with experts, (5) consult with stakeholders, (6) decide what needs adaptation, (7) adapt the original program, (8) train staff, (9) test the adapted materials, (10) implement, and (11) evaluate (see Figure 2 for a detailed description of each category). Steps for adaptation for each Tribal Nation will be discussed within the context of each category below. We do not present steps (10) and (11) as those are reported once the study has been completed.

### Assessing the community needs

The Johns Hopkins Center for Indigenous Health (JHCIH) has a successful three-decade relationship working with federally recognized tribes in the Southwest. JHCIH has a long history of scaling public health studies in reservation and rural communities. JHCIH’s earliest work focused on collaborating with the Navajo Nation, the communities of focus in this study. JHCIH remains committed to addressing elevated rates of mental distress across these Tribal Nations. Over the years, the JHCIH has successfully recruited members of these two tribal communities to serve as research staff and eventually as principal investigators of major studies. This approach has helped researchers to understand the main drivers of poor health and systemic issues that exacerbate infectious diseases and allowed this research team to be well-equipped to shift their focus to supporting the mental health of PSS study participants. This history combined with initial data from the PSS Cohort study led to our team quickly responding to the mental health needs of the Diné and Apache children and adults most in need.

### Understanding and selecting the EBIs

Given the complexities of working within an ongoing pandemic with changing public health mitigation approaches, our team sought brief interventions that were both feasible and impactful. Our teams in both tribal communities have a long history of working on suicide prevention including the development of the award-winning Celebrating Life Program [CL; (13)]. This work combined with Navajo teachings on connections to others which had been undermined by the pandemic, led our team to select interventions that have been shown to reduce distress and social isolation. Our team selected both the Safety Planning Intervention (14) and Caring Contacts (15) interventions as priority evidence-based programs to adapt. Both these interventions are brief and require minimal in-person contact, which was key given the changing nature of the COVID-19 pandemic, particularly on Tribal lands.

### Select the intervention

Safety planning interventions (SPI) are a crucial component of suicide prevention strategies and are considered an evidence-based intervention that has successfully been used with Indigenous communities by researchers at JHCIH (16). The SPI is a brief intervention that directly targets suicide risk with demonstrated efficacy (27). The SPI aims to provide people with an individualized set of steps that can be used progressively to both reduce risk and maintain safety when distressing thoughts emerge. In the SPI, safety plans are developed collaboratively between providers and at-risk individuals. The developers of SPI have also developed and tested an enhanced version of safety planning known as SPI+, which includes a series of brief telephone calls after discharge that focus on assessing risk, revising the safety plan, and facilitating connections to care. The SPI+ is conceptualized to target three proximal targets and underlying mechanisms of suicide risk including increasing coping, decreasing access to lethal means, and increasing engagement in care (17). This enhanced version was utilized in the +CiM study.

Similarly, the Caring Contacts intervention is a low-cost, low-intensity mental health program designed to reduce suicide risk, which involves sending periodic, brief, non-demanding messages of care and concern to individuals who have exhibited suicide risk (18, 19). It has been shown in numerous studies to reduce the risk of suicide and associated thoughts and behaviors (15). The underlying mechanisms thought to drive change include instilling a sense of connectedness and social support, which are well-documented protective factors against mental illness and suicidal thoughts and behaviors. These messages are perceived as a supportive presence that may disrupt feelings of isolation and hopelessness, common factors contributing to suicidal thoughts.

### Consult with experts

Research staff from the CL program were consulted to provide their thoughts and feedback on both the intervention adaptation and study design. Many of the CL staff have nearly 20 years of experience in the mental and behavioral health field, and several serve as expert trainers in suicide prevention interventions. Key questions for experts included, how to broaden the target of these interventions to not only focus on suicide risk but to apply it to broader mental distress. Navajo Nation CAB members provided their thoughts and feedback on the appropriateness of these interventions in their communities at the time. Finally, our team engaged two suicide and mental health experts, both clinical psychologists who have worked closely with Indigenous communities, at the JHCIH, who also provided their feedback on the appropriateness and potential adaptation of the selected interventions.

### Consult with stakeholders

The research team utilized community-based participatory research methods to engage community input. We relied heavily on the parent study’s (PSS) CAB members from the participating communities to provide us with valuable input throughout all stages of the adaptation process for +CiM. We met virtually for CAB meetings with advanced notification to maximize attendance and availability of CAB members. CAB members were members of the tribal nations, community partners, physicians, educators, and parents. For +CiM on the Navajo Nation, we recruited adolescent youth between the ages of 12–16 to serve on our youth advisory board, mostly to aid with the adaptation of our safety plan intervention for youth. Prior to CAB meetings, proposed adaptations of the intervention materials were sent to members for review. During CAB meetings, the project PIs, one of which is a member of the Navajo Nation, facilitated the discussion about the culturally tailoring process and proposed procedures while study coordinators recorded feedback and responses. The PIs and coordinators met during weekly meetings to discuss and synthesize CAB member feedback.

### Decide what needs adaptation

Based on these notes during these CAB meetings, the feedback was discussed as a team and themes were identified based on discussion and direct feedback during these sessions. Cultural responsivity and sensitivity were the primary areas recommended for adaptation, as recommended by CAB members. This included thinking through procedures for how to introduce the concepts of mental health and suicidality with AI/AN people who have cultural taboos against discussions of death and loss of life. In addition, CAB members from both communities suggested the use of culturally tailored messaging for the Caring Contacts materials. Therefore, two distinct types of Caring Contact messages were developed, one set for each tribal community.

### Adapt the original program

The recommendations from the CAB members from both communities were directly incorporated into existing safety plan materials from Stanley and Brown (14). Additional adaptations included the creation of a safety planning guide created specifically for use in reservation-based communities and incorporated cultural safety practices. This included helping participants to connect with not just family members and local and national crisis resources, but also to spaces in their community that make them feel safe (e.g., a traditional dwelling, an outdoor space or a family gathering area). Safety plans were individualized for adolescents and youth and were field tested with members of our community partners and youth advisory council that consisted of Navajo adolescents. Safety plans have been utilized in other community-based suicide and other violence prevention and activities and were adapted through input from local teams that work on suicide prevention in the local communities (20, 21).

### Train staff

After the adequate approvals were obtained from institutional and tribal review boards, the research team went into formal and informal training procedures to train field staff on safety plans which became known as “Coping Plans” for adult participants and “Helping Plans” for youth participants. As timing was a major factor with the study, we opted for using our existing PSS research team who were already trained in responsive research practices and conducting suicide prevention interventions. An in-person meeting was conducted with community-based teams with the project leads training staff on safety planning, standardization of conducting the plan, and methods for data collection. Following the in-person meeting, a community-based investigator provided on-going in person support to teams which included role play with life and retroactive feedback and direct coaching. In addition, training videos were recorded to allow team members access to a pre-recorded standardized training session with a participant.

### Test the adapted materials

Once the study team was trained through in-person role play scenarios that included a structured feedback sessions from study team leads in which a fidelity checklist was used to track key implementation steps, the study team was ready to implement the safety plans with participants. The training fidelity checklist was used as a guide for ongoing fidelity checks to maintain the standardization of safety plan administration. The team then moved into active recruitment and consenting of participants.

## Results

The COVID-19 pandemic revealed many challenges in delivering mental health interventions to vulnerable Indigenous populations. This included the complete halting of in-person programming, limited mental health personnel, and an overworked healthcare system that shifted all attention to pandemic mitigation. What was learned quickly with the adaptation of the safety planning intervention with +CiM was that EBIs can be culturally adapted and modified quickly and effectively to meet the urgent mental health needs of AI/AN communities. In precision public health, we think of how to quickly initiate action to meet the health care needs of a community experiencing inequity. An essential part of providing effective mental health support to reservation-based families on the Diné is understanding the beauty and intricacy of Indigenous connectedness and how disruptions can lead to increased community stress. Using a precision public health approach to determine an area of need and applying an EBI within an Indigenous conceptual framework shows that researchers can effectively respond to community crises in culturally responsive manners. To accomplish this, there must be existing relationships with tribal communities.

### Strong indigenous community partnerships

Strong community partnerships, developed over time and following CBPR processes, in the two tribal communities played a vital role in improving health outcomes and promoting overall well-being in our study participants. This specifically included transparency throughout the research process, which included frequent reporting to CAB members and community partners. Health is not solely determined by access to healthcare services or individual behaviors; it is influenced by a wide range of social, economic, environmental, and cultural factors. By fostering collaboration between healthcare providers, community organizations, local government, and residents, strong community partnerships can address these underlying determinants of health and create sustainable improvements.

### Indigenous community health workers

The community health worker (CHW) model is a strategy that involves trained individuals from within the community who act as a bridge between healthcare providers and the community members they serve (22–24). CHWs are tribal community members who live and work in tribal communities, who are trained to provide basic healthcare services, health education, and support to underserved populations, particularly in rural or disadvantaged communities. When it comes to improving mental health in rural communities, the CHW model can be highly effective. The use of CHWs has proven to be an effective public health approach to addressing mental health needs in these two tribal communities. CHWs establish strong connections with community members, gaining their trust and understanding of their unique cultural, social, and economic contexts. These paraprofessionals engage with individuals and community leaders to identify mental health challenges, stigma, and barriers to care. CHWs act as navigators, helping individuals access mental health services in their communities. They assist with making appointments, accompany individuals to appointments if needed, and help navigate the complex healthcare system. They provide information about available mental health resources, such as counseling centers, support groups, or helplines. CHWs often have similar backgrounds or life experiences as the community members they serve. This shared experience allows CHWs to offer empathetic and culturally responsive peer support, reducing the stigma associated with mental health and creating a safe space for individuals to share their struggles. CHWs can also advocate for the needs of their community, raising awareness among policymakers and healthcare providers about the mental health challenges faced by rural populations. CHWs maintain ongoing contact with individuals to ensure they are connected to appropriate mental health services and to provide continued support and encouragement. They can conduct regular check-ins, reinforce treatment plans, and assist with medication adherence, if applicable. This continuity of care improves treatment outcomes and reduces the risk of relapse.

Overall, the CHW model offers a comprehensive approach to improving mental health in rural tribal communities. By leveraging their local knowledge, cultural sensitivity, and close relationships with community members, CHWs can address the unique challenges faced by rural populations, reduce barriers to care, and promote mental well-being. Care coordination is crucial in mental healthcare as it plays a vital role in ensuring effective and efficient delivery of care to patients. It involves the seamless integration of various healthcare professionals, services, and resources to create a comprehensive and well-coordinated plan of action. By facilitating communication, collaboration, and information sharing among providers, care coordination promotes continuity, avoids duplication of efforts, reduces medical errors, and enhances patient outcomes. It helps to optimize the use of healthcare resources, improve patient satisfaction, and increase the overall quality and safety of care. Ultimately, care coordination serves as a critical link that connects patients to the right care at the right time, making it an essential component of a patient-centered and efficient healthcare system.

## Discussion

During the COVID-19 pandemic, it was apparent early on that the mental health of adolescents and adults would be greatly impacted due to the constant exposure to stress, loss of life, and major disruption to social networks. When working with communities that have experienced elevated stressors linked to a natural disaster or pandemic, the adaptation of mental health-related interventions must take precedence in recovery efforts. This adaptation paper highlights how researchers engaged tribal community member partners can come together to develop solutions to respond to elevated rates of mental health crises in culturally responsive ways. This paper also highlights the importance of building strong working relationships with tribal public health programs, schools, and tribal governments to plan sustainable community-based interventions. There were limitations to implementing safety plan interventions, without the cultural adaptations, on the Navajo Nation. Therefore, we present in this paper a model for how to enhance and adapt exiting EBIs to pilot across Indigenous communities. When there are limited mental health solutions in reservation-based communities, research teams must strategize how to use what works and how to further tailor these solutions to the everchanging needs and demands of Indigenous communities. It is the intent of the researchers to track and report how these EBIs worked on the Navajo Nation and more papers will be published sharing the preliminary findings.

## Data Availability

The original contributions presented in the study are included in the article/supplementary material, further inquiries can be directed to the corresponding author.
